# Experiences of patients and physiotherapists with blended internet-based vestibular rehabilitation: a qualitative interview study

**DOI:** 10.3399/bjgpopen20X101092

**Published:** 2020-10-28

**Authors:** Vincent A van Vugt, Anja JThCM de Kruif, Johannes C van der Wouden, Henriëtte E van der Horst, Otto R Maarsingh

**Affiliations:** 1 Department of General Practice, Amsterdam UMC, Vrije Universiteit Amsterdam, Amsterdam Public Health Research Institute, Amsterdam, The Netherlands; 2 Department of Health Sciences, Faculty of Science, Vrije Universiteit Amsterdam, Amsterdam Public Health Research Institute, Amsterdam, The Netherlands

**Keywords:** general practice, primary health care, dizziness, vertigo, internet-based vestibular rehabilitation, blended care, qualitative research, vestibular symptoms

## Abstract

**Background:**

Internet-based vestibular rehabilitation (VR) with physiotherapy support, known as blended VR, was effective in reducing vestibular symptoms in a recent randomised controlled trial. Blended VR is a complex intervention comprised of physiotherapeutic visits, the vertigo training website, and VR exercises. Because of these interacting components, it is important to understand how blended VR works, for whom it works best, and how it should ideally be delivered.

**Aim:**

To investigate the experiences of both patients and physiotherapists with blended internet-based VR.

**Design & setting:**

A qualitative interview study was performed with patients who received blended internet-based VR with physiotherapy support, and physiotherapists who provided this support.

**Method:**

Semi-structured interviews were conducted with 14 patients and eight physiotherapists after the 6-month follow-up of the randomised trial. All interviews were audio-recorded, transcribed, and thematically analysed.

**Results:**

According to both patients and physiotherapists, the physiotherapist visits were useful in providing personal attention, helping patients safely execute exercises, and improving patients’ adherence to therapy. Some patients said they did not need physiotherapist support and, according to physiotherapists, both the necessity and the optimal way to deliver guidance differed greatly between patients. The Vertigo Training website and exercises provided patients with a sense of control over their symptoms. Patients reported that the VR exercises were easy to perform and most patients continued to use them long after the trial ended.

**Conclusion:**

In blended VR, physiotherapeutic visits appear to offer benefits above the vertigo training website and VR exercises alone. Physiotherapy support may best be used when individually tailored.

## How this fits in

Internet-based VR is a recently developed, exercise-based form of treatment for vestibular symptoms. This qualitative study was embedded in a randomised controlled trial, which showed that automated internet-based VR with physiotherapy support is safe and effective in reducing vestibular symptoms. According to both patients and physiotherapists, the physiotherapist visits provided several benefits on top of the vertigo training website and VR exercises. Ideally, the physiotherapeutic guidance should be tailored to each individual patient and not every patient might require physiotherapy support.

## Introduction

Vertigo and dizziness are common complaints in general practice.^1^ Although the ‘medical’ definitions for vertigo and dizziness differ, patients often use the terms interchangeably.^2^ Vertigo (that is, vestibular symptoms) considerably impacts on daily activities, and is also associated with a substantial economic burden owing to loss of working days, high use of healthcare services, and increased fall risk.^3^ Based on assessment of timing and triggers of vestibular symptoms, patients can be classified as having either an acute vestibular syndrome, episodic vestibular syndrome, or chronic vestibular syndrome.^4–6^ A chronic vestibular syndrome consists of vestibular symptoms lasting months to years, which includes features suggesting persistent vestibular system dysfunction.

VR, an exercise-based treatment consisting of daily graded exercises to stimulate the vestibular system, can effectively reduce vestibular symptoms in vestibular dysfunction.^7,8^ Currently, guidelines in the UK,^9^ US,^10,11^ and The Netherlands^12^ recommend VR to treat chronic vestibular syndromes. In a randomised controlled UK trial, standalone internet-based VR led to less vestibular symptoms compared with usual care.^13^ The standalone VR intervention’s content and functionality was optimised for users by using patients’ experiences.^14,15^ However, exacerbation of symptoms and doubts about the exercises’ effectiveness constituted barriers for implementation.^14,15^

Blended internet-based interventions, where online treatment and contact with healthcare professionals are combined, have shown additional value over standalone internet-based interventions in treating patients with stress, anxiety, and depression.^16–18^ It has been shown in a recent randomised controlled trial that blended internet-based VR with physiotherapy support was more effective than usual care in reducing vestibular symptoms.^19,20^ However, blended VR is a complex intervention comprising physiotherapeutic visits, the vertigo training website, and VR exercises. More information about (the interaction of) these components is needed to understand how blended VR works, for whom it works best, and how it should ideally be delivered. Therefore, a qualitative study was performed to investigate the experiences of patients who received blended VR, and of the physiotherapists who provided this support. The relevance of this study for GPs is substantial, because the inexpensive and easily accessible internet-based VR has the potential to become an (if not the most) important tool in general practice to treat chronic vestibular symptoms. Analysing patients' and physiotherapists' experiences with blended VR will help to guide future implementation strategies.

## Method

A qualitative interview study was carried out with blended internet-based VR participants and physiotherapists, embedded in a randomised controlled trial, which has been described in more detail in previous publications.^19,20^ In short, 322 patients aged ≥50 years with a chronic vestibular syndrome were randomised to receive blended VR, standalone VR, or usual care.^20^ The content of the blended VR intervention is described in Box 1. Follow-up measurements were collected after 3 and 6 months. In the trial protocol, it was planned to interview both patients receiving blended VR and standalone VR to gain insight into different types of internet-based VR.^19^ Because experiences of patients undergoing standalone VR had already been investigated,^14,15^ the research team chose to focus exclusively on blended VR. In addition to blended VR participants, it was decided to also interview physiotherapists who provided support in blended VR, as the physiotherapist viewpoint provides a different perspective. The consolidated criteria for recording qualitative research (COREQ) was followed to conduct this study, and reported the 32-item COREQ checklist in Supplementary Table S1.

Box 1 **. Blended internet-based vestibular rehabilitation with physiotherapy support**

The internet-based vestibular rehabilitation (VR) intervention Vertigo Training consists of 6-weekly online sessions and daily VR exercises. In the first online session, written instructions and video demonstrations are used to teach participants the six core VR exercises. Participants are asked to perform these exercises twice daily for 10 minutes during the intervention period. Every week, the participant scores the level of vestibular symptoms caused by each of the six exercises in a new online session. Vertigo training uses these scores to automatically produce an exercise prescription for the coming week, tailored to the participant’s vestibular symptoms.

In addition to the Vertigo Training website, a trained physiotherapist visits participants twice at home for support. These physiotherapy sessions are planned in weeks one and three of the 6-week intervention period and last for 45 minutes each. During these sessions, the physiotherapist: (a) provides information about the background of vestibular symptoms and VR; (b) elicits and addresses doubts and concerns about vestibular symptoms and VR; (c) teaches the participant how to use the online intervention; (d) describes and takes the participant through the six core VR exercises; (e) advises on how to anticipate and cope with obstacles to adherence; and (f) provides support and encourages adherence. All physiotherapists receive a treatment protocol and are trained by a member of the research team to adhere to this protocol.

### Recruitment

All 14 patients completed the 6-month follow-up period in the blended VR trial arm, and all eight physiotherapists provided blended VR patients with support in the same trial. Purposive sampling was used to select a heterogeneous group of patients and physiotherapists in which at least the following characteristics for both therapists and patients varied: sex, age, and urbanisation. For patients, the study also strived for heterogeneity in intensity of vestibular symptoms, education level, number of completed online sessions, and living arrangements. All selected patients and physiotherapists were contacted by phone to invite them for an interview. If the patient or physiotherapist was interested in participation, they received study information and an informed consent form concerning the qualitative study. The majority of patients who were approached for an interview consented to participate, but four patients declined owing to time constraints. All physiotherapists who were approached for the interview study consented to participate. All patients and physiotherapists provided written informed consent. Recruitment continued until no new themes were identified.

### Interviews

Semi-structured interviews with patients and physiotherapists were conducted in May–June 2018. Most patient interviews took place at the patient's home, and all but one physiotherapist interview was conducted at the physical therapy clinic. The lead author (VvV) interviewed 11 out of the 14 patients, and all of the physiotherapists. A trained research assistant (WK) interviewed the other three patients. VvV is a male researcher and GP specialty trainee, and WK is a female research assistant. The semi-structured interviews were guided by two separate topic lists, one for the patient and one for the physiotherapist interviews, which were based on previous research.^14,15^ During the interviews, the interviewers constantly reviewed the topic list and added relevant items that were identified in preceding interviews. All interviews were audio-recorded and transcribed verbatim.

### Analysis

A thematic analysis approach was carried out according to Braun and Clarke.^21^ The transcripts were read several times and the texts were divided into fragments, and codes were assigned to these fragments (open coding). Subsequently, codes were assigned to themes and, finally, the categories of several transcripts were related to one another (axial coding) using the qualitative software programme ATLAS.ti (version 7). Two authors (VvV and AdK) separately coded the first two transcripts and then compared codes, resolved discrepancies and reached consensus on an initial framework. All the codes were organised into a mind map. The preliminary conclusions based on this mind map were thoroughly discussed between VvV and AdK. The last phase of the analysis was selective coding. This implies that the essence of what each theme is about was identified, relations were searched for through constant comparison across cases, deviant cases were looked for, and variation was analysed within and between cases. Finally, with the help of the different themes, insight was gained into the spectrum of differences and similarities of the results to answer the research question. All findings were discussed in the project team.

Interviews were analysed in Dutch using ATLAS.ti (version 7). After completion, all codes, themes, and quotes were translated into English by the authors.

## Results

Characteristics of the 14 patients and eight physiotherapists are described in Table 1. Three main themes both for patients and physiotherapists were identified: (1) perceived value of physiotherapist visits; (2) content and logistics of physiotherapist visits; and (3) experiences with the vertigo training website and exercises.

**Table 1.** Characteristics of patients and physiotherapists who were interviewed

**Patient**	**Sex**	**Age, years**	**Geographical area**	**Intensity of vestibular symptoms^a^**	**Education level**	**Number of online sessions completed**	**Living situation**
PA01	Female	78	Urban	3 (low)	Low	1	Lives alone
PA02	Female	71	Urban	12 (high)	Low	6	Lives with partner
PA03	Female	55	Urban	19 (high)	High	6	Lives alone
PA04	Female	67	Urban	18 (high)	High	6	Lives with partner
PA05	Female	65	Rural	23 (high)	High	5	Lives with partner
PA06	Female	74	Rural	7 (low)	Medium	1	Lives with partner
PA07	Male	59	Rural	12 (high)	High	6	Lives alone
PA08	Female	57	Suburban	5 (low)	High	6	Lives with partner
PA09	Female	68	Suburban	14 (high)	Medium	6	Lives with partner
PA10	Female	62	Urban	15 (high)	High	6	Lives alone
PA11	Male	72	Urban	15 (high)	Low	6	Lives alone
PA12	Female	62	Urban	5 (low)	High	6	Lives with partner
PA13	Male	53	Urban	16 (high)	High	3	Lives alone
PA14	Female	91	Urban	29 (high)	Medium	1	Lives with partner
**Physiotherapist**	**Sex**	**Age,** **years**	**Geographical area**	**Number of trial patients visited**			
PH15	Male	60	Suburban	5			
PH16	Female	62	Rural	4			
PH17	Male	60	Suburban	6			
PH18	Male	30	Urban	9			
PH19	Female	49	Rural	9			
PH20	Male	27	Urban	16			
PH21	Female	54	Urban	6			
PH22	Female	35	Suburban	6			

^a^Vestibular symptoms measured by Vertigo Symptom Scale – Short Form. A score of 12 points or higher is defined as severe vestibular symptoms.

### Perceived value of physiotherapist visits

Many patients appreciated the opportunity to discuss their vestibular symptoms with the physiotherapist and ask specific dizziness-related questions. Both patients and physiotherapists described this personal attention as an important added value of the visits:

*‘Well, it was just talking about how things were going. Just the interest in me so to speak. It is, well you know, you can talk about it with family but they have no clue what it is if you don’t experience it yourself. To speak with someone who knows how it goes in other people, than that’s nice, just to have a chat about it. Even though it doesn’t directly achieve anything. It is just nice.’* (PA05)

*‘You are always of added value, because just the fact that you give people attention, that they are heard, that they notice that someone takes them seriously, I definitely think that those are important factors. Not just practising the exercises.’* (PH19)

Another important aspect of the physiotherapist visits was help in executing the VR exercises properly. The majority of patients mentioned having received helpful tips, often related to the pace of the exercises:

*‘Because that is what the physiotherapist can do, I mean, I can do a video, I can imitate it, but that does not mean that I am doing it in the same way.’* (PA12)

Almost all physiotherapists also thought they improved the execution of exercises. Sometimes adjustments were minor, but some physiotherapists described patients who had created ineffective variations of the exercises that were very different from the prescribed exercises. Physiotherapists generally found that patients were open to their suggestions, and applied their tips for executing the exercises well. Some patients reported that the physiotherapist paid extra attention to safety, especially when performing exercises with eyes closed or when walking around. Some physiotherapists also mentioned that their support increased the safety of the patients and reduced fear, especially for exercises that had to be performed while walking around. Half of all patients mentioned that they appreciated that the physiotherapist confirmed they performed exercises adequately, and several physiotherapists also said that confirmation was important to patients. Some patients found that the physiotherapist visits added weight to the intervention and made it feel more like a serious treatment:

*‘Otherwise it would just become an internet titbit, like “Oh well, let’s give it a go”.’* (PA10)

More than half of the patients said the physiotherapist visits did not affect their daily adherence to the exercises. They practised every day because they felt a commitment to the treatment. However, some patients said the physiotherapist did help them adhere to treatment. Because they knew the physiotherapist would come to visit them again later, they felt an extra incentive to keep practising daily. On the other hand, all physiotherapists thought they had a positive influence on adherence to therapy. According to many physiotherapists, patients felt held accountable because they knew the physiotherapist would visit them again. One physiotherapist described that the effect on adherence to therapy was caused by a combination of factors:

*‘You could say the overall effect is that compliance to therapy is improved. You take away part of the fear, which makes patients practice better, or you provide patients with a better understanding of the exercises, what actually makes patients perform the exercises more often.’* (PH16)

A few physiotherapists mentioned providing computer assistance as an important aspect of their visits. However, most physiotherapists did not access the computer during the visits and advised patients to simply contact the research team. None of the patients mentioned computer assistance as an added value of the physiotherapist visits.

The majority of patients said they had no negative remarks on any aspect of the physiotherapist visits. Some patients felt the physiotherapist visits provided little additional value over the vertigo training website. According to several patients, the exercises were clear enough and quite easy to do by following the instructions on the website. On the other hand, all physiotherapists said they thought their visits had beneficial effects on top of an internet-based treatment. Most physiotherapists did mention that the level of physiotherapy support that patients required was not the same for everyone:

*‘Well, at my first visit I just asked, how are you, did you already perform the exercises? Well, then they showed me. In one case, this woman, that is really the best example, she actually knew the exercises by heart and I only had to observe, and think, well what am I even doing here?* [Laughs] *And looking at the last woman I saw, yeah she really had no clue what to do.* […] *she even had trouble starting the computer, and where to look in the computer, and she didn’t know if she would get new exercises or not. So it was all not very clear to her.’* (PH19)

Most physiotherapists described clear differences between patients in the way they handled the computer, executed the exercises, needed confirmation, and appreciated the physiotherapist visits. Older age, severe comorbidity, a high intensity of vestibular symptoms, cognitive problems, and trouble with the Dutch language were all mentioned as reasons that increased the need for physiotherapy support.

### Content and logistics of physiotherapy visits

The content of the first and second visit was described in the same manner by both patients and physiotherapists. In the first visit the physiotherapist talked with patients about their vestibular symptoms, provided information about vestibular symptoms and the exercises, answered questions, asked patients to show them how they performed the exercises, and corrected them if needed. In the second visit, the physiotherapist asked patients to demonstrate the exercises again to check if they had implemented the improvements suggested in the first visit. Because exercises were often more difficult at this stage (for example, performed while walking around), the physiotherapist sometimes offered support and gave tips to practise safely. The physiotherapist also asked about the adherence to therapy in the second visit. All physiotherapists said they followed the trial protocol for all patients. Several physiotherapists were sometimes tempted to deviate from protocol in patients with specific vestibular conditions, such as benign paroxysmal positional vertigo, or invalidating comorbidity, such as Parkinson’s disease that, according to them, required a more personalised treatment.

Both patients and physiotherapists mentioned that the second visit was often much shorter than the first visit. The first physiotherapist visit mostly took place in the first week, and the second visit in the third week of the 6-week treatment period. Both patients and therapists appreciated the timing of the first visit, including the fact that patients had already started practising the exercises. Opinions about the optimal timing of the second visit were more varied, most patients found it hard to remember when the second visit exactly took place. None of the patients would have appreciated the visit to occur sooner than the third week, but several physiotherapists would have liked a visit in the second week for certain patients to be sure the exercises were executed properly. Some patients and physiotherapists said the second visit could have been planned a little later. That way more patients would have reached the more difficult walking exercises, and would benefit more from the physiotherapist’s guidance. Most patients and physiotherapists were satisfied with two physiotherapeutic visits. For several patients one visit would have been enough, and some physiotherapists also said that this would suffice for patients who performed well. None of the patients thought they needed a third visit. However, many physiotherapists thought that certain patients might have benefited from more than two visits:

*‘I think for one-third of the patients, I would be able to deal with them in one visit. Certainly. Yes*. [Interviewer: And the other two-thirds?] *Those were the older patients*. [Interviewer: And you would rather visit them three times?] *Yes, yes, exactly.’* (PH22)

Patients generally found the home visits convenient and pleasant. Physiotherapists also mentioned several advantages of home visits. The home situation felt familiar to patients and physiotherapists could observe patients in their natural habitat to assess if they had created a safe environment to perform their exercises:

*‘Advising someone “Place a chair in front of you, and practise close to the sofa, so if you go backwards, right, then nothing can go wrong”. And that gave them the confidence, and they could just do it. “Oh yeah, well, I’ll do it like this then.” Yeah, yeah, and you might not have figured that out here in the centre. That, at home, it is like this.’* (PH18)

A disadvantage of home visits according to physiotherapists was that it was more time-intensive than treatment in the clinic and, therefore, more costly. Although patients enjoyed the home visits, some said they could have just as easily visited the physiotherapy clinic. Several physiotherapists said that home visits were most valuable in older patients with intense vestibular symptoms, while most other patients could just as well have been seen at the physiotherapy clinic.

### Experiences with vertigo training website and exercises

Patients generally appreciated the information provided, the exercise videos and the possibility to print out instructions for the exercises on the vertigo training website. One patient found the website difficult to navigate and not user-friendly.

All patients found the exercises easy to perform and implement in their daily life. Patients mentioned that the exercises worked best when they did them each day. Physiotherapists also noticed that most patients performed their exercises twice daily. Most patients were not anxious to perform any of the exercises, but a few patients were slightly anxious to perform the walking exercises. None of the patients or physiotherapists described any serious side effects, most only mentioned a temporary increase in dizziness. One patient even welcomed the dizziness and nausea she experienced after performing the exercises, because it made her feel she was doing something that works. The exercises consisted of neck movements, and both patients and physiotherapists talked about the effects it had on their neck. At first, practising often led to pain in the neck, but after a couple of weeks patients felt their neck was more flexible than before. Owing to the simple nature of the exercises, many patients doubted at the start of the trial if the exercises would work. Nevertheless, most patients expressed that the exercises had helped reduce their vestibular symptoms:

*‘I think it is surprising that something so simple, can have such an impact! Yeah, it totally surprised me. Because I thought it was all connected to complicated organs, that don’t, I don’t know, function properly. And that it is beyond your influence, it is part of getting older, or having bad luck, but it actually isn’t all that bad.’* (PA01)

The majority of patients said the Vertigo Training website had impacted their life in a positive way. Many patients said it increased their self-confidence. Patients developed more awareness of their vestibular symptoms, which helped give them a sense of control. They learnt what factors exacerbated their symptoms and acknowledged they had subconsciously started avoiding certain activities, such as turning their head in the car or walking in the dark. Several patients mentioned that the exercises gave them control over their symptoms, which they had not previously experienced:

*‘A couple of weeks ago, it came back for a little while, and then I thought, well let’s just do them again. And it wasn’t like I immediately started practising daily, but it was a moment of, just getting back into it, checking how it’s going. So that provides me with a bit of grip. … But I think, well … I can do something about it myself. And of course you never had that before.’* (PA09)

Many patients mentioned that they had continued to do the exercises long after the trial period. Although they were only asked to practise daily for the 6-week intervention period, the majority of patients still used the exercises in some way more than 7 months later. Most patients said they would start the exercises again when experiencing a return or increase of their vestibular symptoms. One patient, who was aged 91 years, said that she continued with the exercises after the intervention period because it took her longer to reach her treatment goals:

*‘Well, so then I had to repeat those exercises until they did not make me dizzy anymore. So before I finally got it right, because that took a long time, certainly a couple of months, before I got the hang of it. Before I could say, “Well, I can stand in the bathroom, without falling” and stuff like that. So that, that takes a very long time! Because look, you are of course old and things don’t go as fast as you would like.* [Laughs]*’* (PA14)

Almost half of the physiotherapists started using the exercises with other patients in their own practice after the trial. Several patients advised the research team to implement the vertigo training website and exercises in daily general practice as soon as possible:

*‘Well, I think it would be nice, because for me it really, really, yeah improved the situation, if it would be more accessible to other people because it is not a, really, heavy treatment. And I, I personally much prefer it over pills against dizziness or whatever.’* (PA10)

## Discussion

### Summary

According to patients and physiotherapists, the physiotherapist visits were useful in providing personal attention, helping patients safely execute exercises properly, and improving their adherence to therapy. Some patients questioned the necessity of physiotherapist support, and physiotherapists also acknowledged that the need for support greatly differed between patients. The optimal amount, timing and place of the visits, therefore, differed for each patient. Patients and physiotherapists were generally positive about the other components of blended VR: the vertigo training website and the exercises. Using the website and its exercises gave patients a sense of control over their vestibular symptoms. They found the exercises easy to perform and implement in daily life. Surprisingly, many patients continued to use the exercises long after the end of the 6-week treatment period and several physiotherapists started using the exercises in their own clinic after the trial. This study provides an important insight for future implementation in general practice: for optimal delivery of blended VR, the number and timing of physiotherapy home visits should be tailored to the individual patient.

### Strengths and limitations

A major strength of the study is that both patients who received blended VR and the physiotherapists who provided this guidance were interviewed. By assessing the situation from these two viewpoints, a comprehensive overview of blended VR was developed. Another strength is the diverse sample of patients and physiotherapists. A limitation of the study is the timing of the interviews. To minimise interference with the randomised controlled trial, the interviews were conducted after patients had concluded the 6-month follow-up period and physiotherapists had finished treating their last patients. This might have made it hard for patients and physiotherapists to remember certain details about the blended VR treatment. Nevertheless, it did provide new insights in the long-term influence of blended VR on patients. Of course, only patients and physiotherapists who were involved in the randomised controlled trial were interviewed. They may have been more motivated to receive exercise-based treatment. The expressed views on blended VR may, therefore, be different from patients who receive blended VR in daily general practice.

### Comparison with existing literature

This is the first study to investigate experiences with blended VR. Patients’ positive opinions about the vertigo training website and exercises were in line with the results of interviews with patients who used the standalone English version of vertigo training (balance retraining).^14,15^ The value of physiotherapy support in providing personal attention and helping to adhere to treatment mentioned in the study was also expressed in a previous qualitative study with patients who received booklet-based VR with telephone support.^22^ An in-depth analysis of this telephone support showed that healthcare professionals were able to build strong therapeutic relationships with patients who are dizzy by using positive communication behaviours such as encouragement, approval, and reassurance of safety.^23^ Physiotherapists in the present study mentioned these elements as the mainstay of their visits. The appreciative way patients described their physiotherapists indicates that a therapeutic relationship also developed in the study. Similarly to the present study, patients who received booklet-based VR without therapeutic guidance reported that VR increased their awareness and made them less insecure about their dizziness.^22^ The sense of control expressed by patients in the present study could, therefore, also have been related to VR itself and not to the therapeutic guidance.

For stress, anxiety, and depression, blended internet-based interventions have produced better treatment results than standalone internet-based interventions.^16–18^ A recent systematic review showed that there is insufficient evidence to say the same is true for chronic somatic disorders.^24^ The randomised controlled trial, in which this qualitative study was embedded, showed that both standalone and blended VR are effective treatments to reduce vestibular symptoms in general practice.^20^ The views expressed by patients and physiotherapists in this qualitative study, indicate that some patients may need more support than others. Determining which patients are likely to benefit more from blended VR over standalone VR is, therefore, essential. This answer may be provided by combining results from this qualitative study with quantitative data. Therefore, a predictive model study is currently underway, which will assess which patient characteristics are associated with treatment success in blended VR and standalone VR.^19^ Possible factors identified in these interviews will be used as potential predictors. Costs may also be important in deciding treatment with blended VR or standalone VR in the future. Both treatments are inexpensive, but blended VR (€166 per patient) is more costly than standalone VR (€39 per patient). An economic evaluation in progress, conducted alongside this randomised controlled trial, will help determine which treatment is more cost-effective.^19^

### Implications for research and practice

The qualitative study indicates that, according to both patients and physiotherapists, physiotherapeutic visits in blended VR have benefits that are not provided by the vertigo training website and VR exercises alone. However, the need and optimal form to deliver physiotherapy support does seem to differ between patients. Just like VR itself, physiotherapy support in internet-based VR should be tailor-made. Luckily, this 'personalised medicine', in which the individual patient's personal characteristics, behaviours, and environment are all taken into account to decide optimal treatment, is already the default approach in general practice medicine.^25^ Quantitative research may help assess which patients require blended VR instead of standalone VR. Mentioned reasons for needing blended VR identified in this study (for example, older age and severe comorbidity) could serve as a starting point for these new studies. To decide the optimal form of physiotherapy support when choosing blended VR, letting physiotherapists decide the location and number of visits needed independently may be the most practical solution. Although, the physiotherapy support was standardised in this trial, this is not how physiotherapists normally deliver personalised support. Evaluating effects and costs when physiotherapists get more autonomy could be the next step in optimising physiotherapy support in blended VR.

The positive patient experiences described in this study further reinforce the position that internet-based VR deserves to be implemented on a larger scale in general practice. The choice to implement standalone VR, blended VR or both in general practice is difficult and will also be dependent on the results of the cost-effectiveness analysis. The findings will help practising GPs to deliver personalised medicine in patients with chronic vestibular symptoms.
